# Characterization of Patients with Non-Small Cell Lung Cancer Using Machine Learning Tools: Relationship with Prognostic Biomarkers and Overall Survival—A Pilot Study

**DOI:** 10.3390/jcm15166439

**Published:** 2026-08-20

**Authors:** Irene Lojo-Rodríguez, Manuel Casal-Guisande, Maribel Botana-Rial, Cristina Ramos-Hernández, Virginia Leiro-Fernández, Almudena González-Montaos, Cristina Pou-Álvarez, Alberto Fernández-Villar

**Affiliations:** 1 Pulmonary Department, Hospital Ribera Povisa, 36211 Vigo, Spain; irene.lojo@gmail.com; 2 NeumoVigo I+i Research Group, Galicia Sur Health Research Institute (IIS Galicia Sur), SERGAS-UVIGO, 36312 Vigo, Spain; maria.isabel.botana.rial@sergas.es (M.B.-R.); cristina.ramos.hernandez@sergas.es (C.R.-H.); virginia.leiro.fernandez@sergas.es (V.L.-F.); almudena.gonzalez.montaos@sergas.es (A.G.-M.); cristina.pou.alvarez@sergas.es (C.P.-Á.); alberto.fernandez.villar@sergas.es (A.F.-V.); 3 Doctoral Programme in Epidemiology and Public Health, International Doctoral School, University of Santiago de Compostela, 15782 Santiago de Compostela, Spain; 4 Department of Design in Engineering, University of Vigo, 36208 Vigo, Spain; 5 Centro de Investigación Biomédica en Red, CIBERES ISCIII, 28029 Madrid, Spain; 6 Pulmonary Department, Hospital Álvaro Cunqueiro, 36312 Vigo, Spain; 7 Department of Functional Biology and Health Sciences, University of Vigo, 36310 Vigo, Spain

**Keywords:** artificial intelligence, machine learning, clustering, lung cancer, biomarkers, prognosis

## Abstract

**Background/Objectives:** Most cases of non-small cell lung cancer (NSCLC) are diagnosed at advanced stages, where prognosis remains poor. Machine learning (ML) offers new opportunities for patient stratification. This study aimed to identify distinct subgroups of patients with advanced-stage NSCLC using unsupervised ML techniques and to evaluate their association with survival outcomes and biomarker expression. **Methods:** 400 patients with advanced-stage NSCLC were analyzed using the k-prototypes algorithm. Clinical, demographic, and analytical variables, including smoking history and comorbidities, were incorporated. Identified clusters were compared in terms of molecular biomarkers and overall survival. A multivariable Cox proportional hazards model was performed to assess the association between cluster membership and overall survival. **Results:** Five patient profiles were identified. Cluster F, characterized by a predominance of women, relatively low smoking exposure, and a higher frequency of epidermal growth factor receptor (EGFR) mutations, showed the most favorable survival profile. Cluster S comprised mainly male heavy smokers with poorer performance status and high metastatic burden, whereas Cluster Y consisted predominantly of younger men without comorbidities but with frequent M1c disease. Clusters E and O showed intermediate outcomes and were characterized by older age with pleural effusion and by an older predominantly male smoking profile, respectively. In the multivariable Cox model, compared with Cluster F, a higher risk of death was observed for Cluster S (HR 1.62, 95% CI 1.01–2.60; *p* = 0.045) and Cluster Y (HR 1.55, 95% CI 1.09–2.21; *p* = 0.015), although the association for Cluster S should be interpreted cautiously. Differences in molecular biomarker distribution were also observed across clusters, particularly for EGFR mutations and programmed death-ligand 1 (PD-L1) expression. **Conclusions:** In this single-centre retrospective pilot study, unsupervised ML identified distinct patient profiles associated with differences in survival outcomes and molecular characteristics. These findings support the potential of data-driven approaches to characterize heterogeneity in advanced-stage NSCLC, although external validation is required before clinical application.

## 1. Introduction

Lung cancer is one of the leading causes of oncological mortality worldwide, representing a medical and scientific challenge. Among the various histological subtypes, non-small cell lung cancer (NSCLC) is the most prevalent, accounting for approximately 85% of cases and constituting the primary cause of cancer-related death [1]. NSCLC is often diagnosed at advanced stages due to the absence of specific symptoms in its early phases [2]. The most well-established risk factors include smoking, exposure to environmental carcinogens such as asbestos or radon, and genetic predisposition [3]. However, not all patients are smokers, and in the specific case of adenocarcinoma, a higher incidence has been observed in non-smoking women [4]. This epidemiological pattern underscores the need to explore the clinical and molecular mechanisms underlying carcinogenesis in this disease [5].

Multiple prognostic factors have been assessed in NSCLC, in addition to the TNM classification [6], which may influence disease progression and clinical outcomes [7]. One of those factors is the Charlson Comorbidity Index, a tool used to estimate the impact of concomitant diseases on patient prognosis [8]. It has been observed that patients with advanced comorbidities have lower rates of chemotherapy administration [9]. Another key parameter for patient characterization at the time of diagnosis is the Eastern Cooperative Oncology Group (ECOG) performance status, which is widely validated as a predictor of survival in oncological patients [10]. Particularly relevant are analytical biomarkers, as studies have shown that patients with brain metastases present significantly lower platelet levels compared to those without central nervous system dissemination [11].

The development of targeted therapies and immunotherapy has significantly transformed the management of NSCLC [12]. Targeted drugs directed at specific genetic mutations, such as epidermal growth factor receptor (EGFR) mutations and anaplastic lymphoma kinase (ALK) gene translocations, have improved both survival and quality of life for many patients. Additionally, the introduction of immune checkpoint inhibitors has contributed to prolonged survival in advanced stages of the disease. However, these therapeutic innovations also highlight the critical need for early diagnosis to maximize their effectiveness and improve patient prognosis.

In this context, artificial intelligence (AI) [13] offers new opportunities to address several challenges in lung cancer. AI-based approaches have shown promising applications in screening, pathological diagnosis, prognosis assessment, and treatment decision support [14]. However, machine learning (ML), particularly unsupervised approaches, remains comparatively underexplored for identifying latent patient subgroups and characterizing disease heterogeneity.

In other respiratory diseases, including chronic obstructive pulmonary disease (COPD), alpha-1 antitrypsin deficiency, and sleep apnea, AI has been used to identify clinically meaningful patient subgroups, supporting disease characterization and healthcare management [15,16,17,18,19]. AI has also been applied to exacerbation prediction and to the diagnosis and classification of other prevalent respiratory disorders [20,21,22,23,24], illustrating its potential for studying heterogeneous clinical populations.

Despite therapeutic and diagnostic advances, many NSCLC cases are still diagnosed at metastatic stages, and substantial heterogeneity exists even among patients with advanced disease [25]. NSCLC can therefore no longer be characterized simply as a disease of elderly smokers, highlighting the need for approaches capable of integrating multiple clinical and biological characteristics to identify distinct patient profiles.

In lung cancer, AI applications have focused primarily on diagnostic tasks, particularly pulmonary nodule detection and malignancy prediction [26]. Other approaches have explored the prediction of molecular alterations such as EGFR mutations and responses to specific treatments [27]. In contrast, validated unsupervised ML approaches integrating routinely available clinical and analytical variables for patient-level stratification in advanced NSCLC remain limited, supporting further investigation in this setting.

To address this need, the primary objective of this pilot study was to identify distinct patient profiles among patients with advanced-stage NSCLC using unsupervised ML approaches and a comprehensive dataset including clinical information, analytical variables, and risk factors. Additionally, we aimed to analyze the association of these profiles with the distribution of key prognostic biomarkers and overall survival.

## 2. Materials and Methods

### 2.1. Study Cohort

The data collected in this study included 400 patients obtained from a retrospective cohort of patients with cytological or histologically confirmed NSCLC between 1 January 2018, and 31 December 2022. The study included patients with locally advanced or metastatic stages (IIIA, IIIB, IIIC, IV) for whom biomarker testing had been requested. Only patients from the dedicated lung cancer specialized consultation at Álvaro Cunqueiro Hospital in Vigo (Galicia, Spain) were included. All cases were presented at a multidisciplinary tumor board specializing in thoracic malignancies to determine therapeutic management according to the guidelines available at the time of diagnosis [28].

Exclusion criteria included patients with NSCLC in localized stages and tumors of other origins.

A systematic collection of information was performed through a review of electronic medical records. A summary of the variables is presented in the first column of Table 1 and Table 2.

The variables included in the clustering analysis, as shown in Table 1, comprised demographic factors (age, sex, smoking status, pack-years, and exposure to cancer-related risk factors), clinical characteristics (symptomatology), and prognostic scales (Charlson Comorbidity Index and ECOG performance status). Several laboratory parameters were recorded at the time of NSCLC diagnosis, including hemoglobin levels, leukocyte count, plasma albumin levels, and total protein levels. Tumor staging was assessed according to the TNM classification [6], and histological subtypes were categorized as adenocarcinoma, squamous cell carcinoma, adenosquamous carcinoma, and non-squamous/undifferentiated carcinoma.

Additional variables collected but intentionally not included in the clustering analysis, as detailed in Table 2, comprised specific driver mutations susceptible to targeted therapy, including EGFR mutations, ALK rearrangements, and proto-oncogene ROS1 mutations, as well as programmed death-ligand 1 (PD-L1) expression [29,30]. These molecular variables were excluded from cluster generation because one of the objectives of the study was to evaluate whether patient profiles derived from demographic, clinical, and analytical characteristics were subsequently associated with distinct molecular patterns. Their exclusion therefore allowed biomarkers to be used as external variables for the biological characterization of the identified clusters, avoiding their direct contribution to cluster definition. Finally, overall survival in months from the time of NSCLC diagnosis and all-cause mortality were recorded.

### 2.2. Study Design

**Figure 1:**
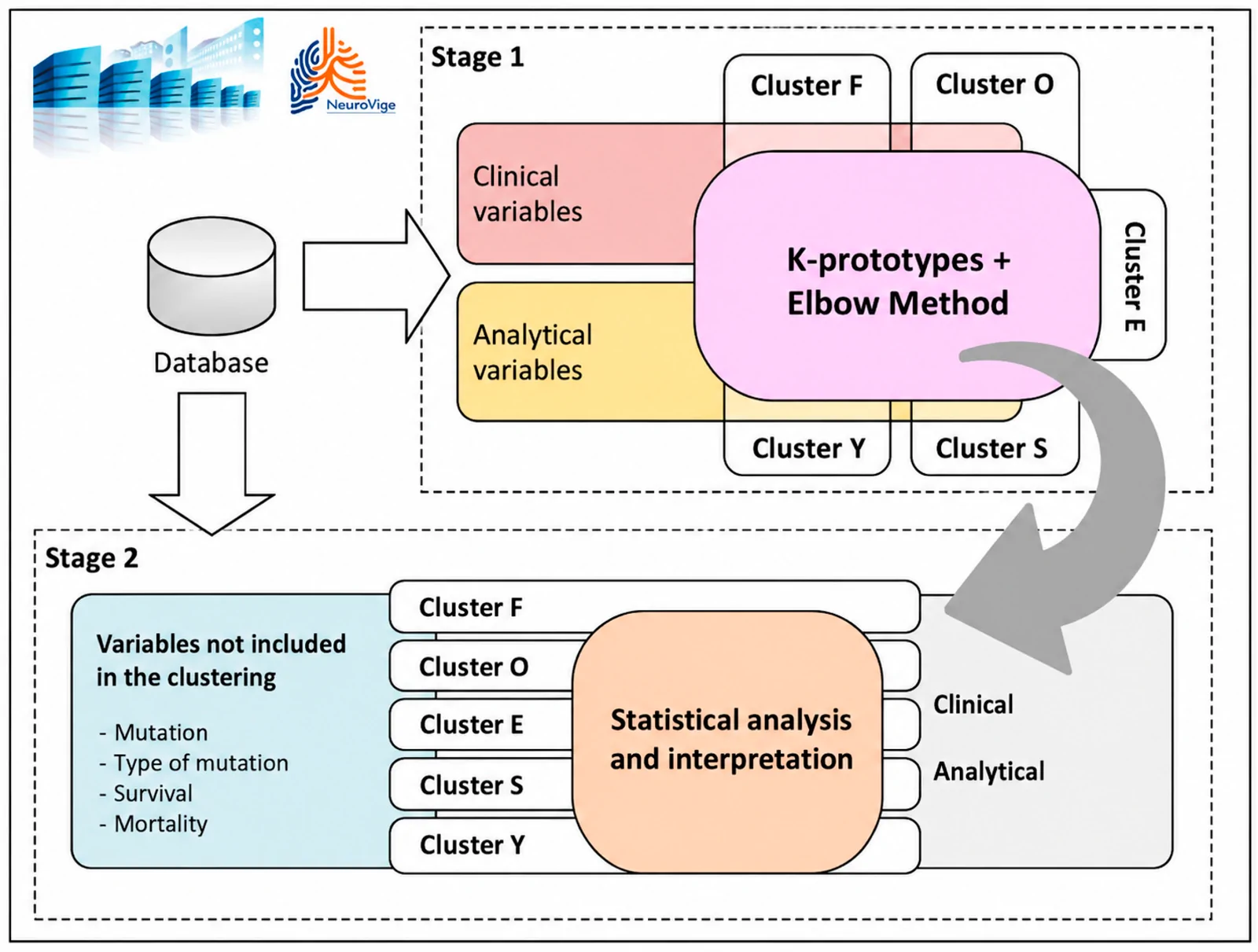
Study methodology. The analytical workflow is structured into two main stages, preceded by a data preprocessing step. During preprocessing, continuous variables were assessed for skewness and transformed when appropriate, followed by normalization to ensure comparability across features, while categorical variables were incorporated directly. In Stage 1, unsupervised clustering of the cohort was performed using the k-prototypes algorithm, allowing the integration of mixed data types. The optimal number of clusters was determined through a combination of the elbow method and internal validation metrics. In Stage 2, a comparative analysis was conducted to evaluate clinical, biological, and outcome differences among the identified clusters using appropriate statistical methods. Survival analyses, including Kaplan–Meier estimation and multivariable Cox regression, were also performed to assess the prognostic relevance of cluster membership.

Figure 1 presents a conceptual overview of the study methodology, structured into two main stages.

Prior to Stage 1, data preprocessing was performed. The distribution of continuous variables was evaluated using the skewness coefficient (Fisher–Pearson method) [31] to identify potential asymmetries that could bias distance-based clustering. Continuous variables exhibiting a skewness coefficient greater than 0.5 were transformed using the Box–Cox method [32], after ensuring positivity of values. The threshold of 0.5 was defined a priori as a pragmatic criterion to identify at least moderately skewed distributions and was not empirically optimized in the present dataset. This preprocessing step was intended to reduce meaningful distributional asymmetry before clustering. Subsequently, all continuous variables were rescaled using min–max normalization to a common range [0, 1], thereby preventing any single variable from disproportionately influencing the clustering process.

Categorical variables were included as categorical inputs, allowing the algorithm to handle them using dissimilarity measures based on category matching, without requiring additional transformation. All patients had complete data across the selected variables.

In Stage 1, cohort clustering was performed. Given the mixed nature of the dataset (continuous and categorical variables) [33,34], the k-prototypes algorithm [35] was selected as it enables a unified distance computation combining numerical and categorical information. To determine the optimal number of clusters, multiple solutions were evaluated across a range of k values (from 2 to 10) using the elbow method [36] applied to the model’s cost function. This selection was further supported by internal validation using the silhouette index [37], computed based on the Gower distance matrix [38]. Based primarily on the elbow and silhouette analyses, the five-cluster solution was selected as the most appropriate statistical solution, with clinical interpretability and internal consistency considered as complementary criteria.

To further assess the internal stability of the selected clustering solution, a repeated subsampling analysis was performed. A total of 500 random subsamples containing 80% of the study population (*n* = 320) were generated without replacement. For each subsample, the k-prototypes algorithm was re-fitted using the same variables and model specifications, and agreement with the corresponding full-cohort clustering assignment, restricted to the patients included in each subsample, was quantified using the adjusted Rand index (ARI) [39]. As a sensitivity analysis, this procedure was applied to the selected five-cluster solution and to the immediately adjacent four- and six-cluster solutions. For the selected five-cluster solution, cluster-specific stability was additionally assessed using the maximum Jaccard similarity between each original cluster and the clusters obtained in each subsampled solution.

As an additional exploratory step, UMAP (Uniform Manifold Approximation and Projection) [40] was applied to the precomputed Gower distance matrix (metric = ‘precomputed’), with n_neighbors = 30, min_dist = 0.3, n_components = 2, and random_state = 42. The resulting two-dimensional projection was used exclusively for visualization and did not inform cluster selection.

In Stage 2, a comparative analysis of the identified clusters was conducted, incorporating the variables listed in Table 2. For continuous variables, overall differences among clusters were first assessed using the Kruskal–Wallis test [41]. When statistically significant differences were detected, pairwise comparisons were performed using the Mann–Whitney U test [41] with adjustment for multiple comparisons using the Bonferroni correction. For categorical variables, overall differences were assessed using the chi-square test [41]. When statistically significant differences were found, pairwise chi-square comparisons with Bonferroni correction were additionally performed. With five clusters, up to 10 pairwise comparisons were possible for each variable, and the Bonferroni correction was applied across the pairwise comparisons performed for that variable. All statistical tests were two-sided, and a significance level of 5% was established for all analyses. Continuous variables are reported as mean and standard deviation, whereas categorical variables are presented as percentages with corresponding 95% confidence intervals, estimated using the Wilson method.

Overall survival was initially explored descriptively using Kaplan–Meier curves according to cluster assignment. Subsequently, a multivariable Cox proportional hazards model was fitted to evaluate the association between cluster membership and overall survival, adjusting for age, sex, and Charlson comorbidity index. The proportional-hazards assumption was assessed using Schoenfeld residual-based tests for age, sex, Charlson comorbidity index, ECOG performance status, and each cluster contrast. ECOG was the only covariate showing evidence of non-proportional hazards and was therefore included as a stratification variable in the final model. The proportional-hazards assumption was subsequently reassessed for all remaining covariates in the final stratified model. In the final analysis, Cluster F was selected as the reference category because it showed the most favorable survival profile in the descriptive analysis.

The study was conducted using Python (version 3.10.13). Clustering analysis was performed using the k-prototypes algorithm implemented in the kmodes library (v0.12.2). UMAP was implemented using the umap-learn package (v0.5.9.post2). Statistical analyses were conducted using SciPy (v1.15.3), and confidence intervals for proportions were calculated using the Wilson method implemented in statsmodels (v0.14.4). Survival analyses, including Kaplan–Meier estimation and Cox proportional hazards modelling, were performed using the lifelines library (v0.30.3).

## 3. Results

### 3.1. Study Population

A total of 400 patients were analysed. Of these, 70.50% were male, with a mean age of 67.29 ± 10.67 years. The average smoking history in the sample was 31.26 ± 27.76 pack-years. Regarding prognostic scales, the mean Charlson Comorbidity Index was 0.74 ± 1.14, while the mean ECOG performance status score was 1.19 ± 0.89.

**Figure 2:**
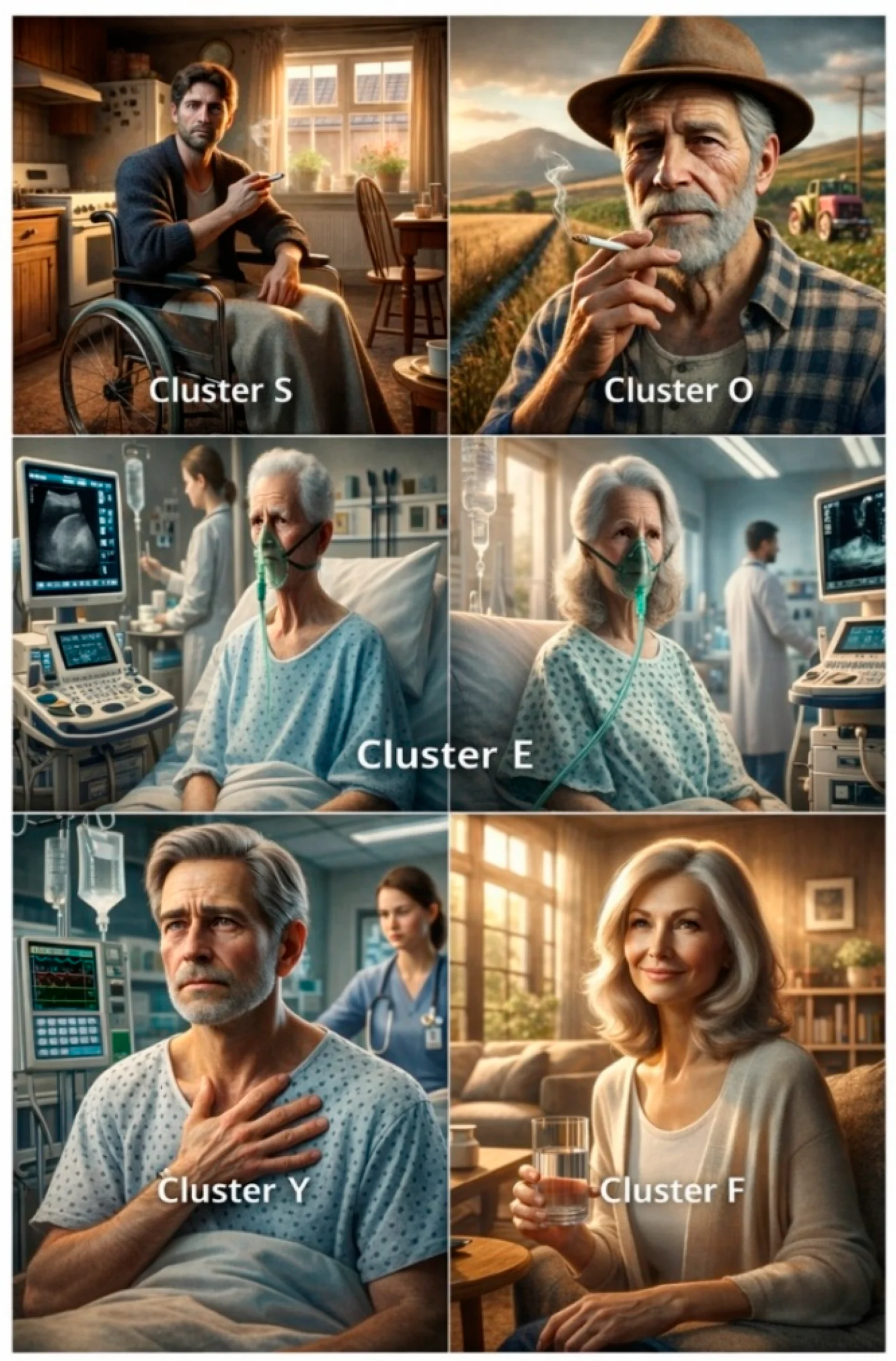
Synthetic illustrative representation of the patient profiles corresponding to clusters S, O, E, Y, and F. Each panel depicts an illustrative clinical scenario based on the aggregate characteristics of the corresponding cluster described in Table 1. Images were generated using ChatGPT 5 (OpenAI) after completion of the clustering analysis and were used exclusively as a visual aid. No real persons are depicted, and the images do not represent individual patients or individual-level study data. The figure was not used for cluster generation, statistical analysis, or clinical inference.

Clustering was performed using the variables listed in Table 1, resulting in five clusters: Cluster F (*n* = 102), Cluster O (*n* = 60), Cluster E (*n* = 42), Cluster S (*n* = 69) and Cluster Y (*n* = 127). To facilitate the interpretation of the identified clusters, a synthetic graphical representation of the aggregate patient profiles was generated after completion of the clustering analysis (Figure 2) using ChatGPT 5 (OpenAI). This figure was created exclusively as an illustrative aid and was not used in cluster generation, statistical analysis, or clinical inference.

A detailed description of each cluster is presented below.

- Cluster S—Smoker Men with poor PS and lack of mutations: This cluster is formed by 17.3% of the sample, with a total of 69 patients, primarily men with a high smoking history. It is characterized by the presence of multiple comorbidities and a worse ECOG performance status, which is the highest in the study. Additionally, their albumin and hemoglobin levels are low, while total leukocyte count is elevated. Pleural effusion is moderately present (27.5%); however, their metastatic burden is much higher than in the other groups, with 85.5% presenting with M1c disease. Similarly, although the most frequent histology is adenocarcinoma, there is a non-negligible proportion of undifferentiated carcinoma (10.1%).
- Cluster O—Older men without clinical or analytical alterations: This second group consists of 60 patients (15%), primarily composed of male smokers with a significant smoking history. The average age in this cluster is 71.3 years. The presence of risk exposures for NSCLC was particularly notable, being the highest among all groups. Regarding analytical parameters, values remained within normal ranges. Although the prevalence of chronic diseases was moderate, the mean ECOG performance status score was among the lowest, and symptom burden was lower compared to the other clusters. M1b disease predominated (70.0%), corresponding to a single extrathoracic metastatic site.
- Cluster E—Older patients with Pleural Effusion and comorbidities: This cluster is smaller, comprising 42 patients (10.5%). It has the highest mean age, approximately 72 years. It consists of both men and women with minimal smoking history but a significant burden of chronic diseases and a high ECOG score. Regarding analytical parameters, this group exhibits mild anemia, with slightly reduced protein and albumin levels. A defining characteristic of this cluster is the high prevalence of pleural effusion (64%), which was almost the exclusive form of metastasis in 59% of cases. The most frequent histological subtype remained adenocarcinoma.
- Cluster Y—Young men without comorbidities and PD-L1 presence: This cluster is the largest, including 127 (31.7%) patients, and it is also the youngest group, with a mean age below 65 years, predominantly male. Regarding smoking history, their levels are close to the average of the sample. However, this group exhibits the highest symptom burden and has a moderate ECOG performance status, although no comorbidities are present. In terms of analytical parameters, they do not show significant deviations in protein, hemoglobin, or albumin levels compared to the population mean, but their leukocyte count is elevated. This group also showed a high prevalence of M1c disease (79.53%) and a notable proportion of undifferentiated tumors.
- Cluster F—Predominantly female patients with low smoking exposure and higher EGFR mutation frequency: This cluster consists of 102 patients (25.5%). Women represented 61.76% of this cluster, which was also characterized by relatively low smoking exposure. It is the second youngest group. Regarding analytical variables, albumin levels were close to the overall mean, while total protein levels were slightly lower than the overall mean. However, anemia is not present, and their total leukocyte count remains within the normal range. Patients in this cluster do not present associated comorbidities, and their performance status is among the lowest across the different clusters. The predominant histological subtype in this group is adenocarcinoma, with a higher frequency of EGFR gene mutations.

To evaluate the robustness and clinical relevance of the clustering solution, both quantitative metrics and visual inspection were employed. These analyses were aimed at assessing the internal consistency of the selected five-cluster model and its ability to capture clinically meaningful patient stratification.

The average silhouette index for the five-cluster solution was 0.13, computed using the Gower distance matrix. Although this value indicates limited separation among clusters, it is not unexpected in real-world clinical datasets, which are often highly heterogeneous and include mixed-type variables. In such contexts, cluster boundaries are usually not sharply defined, and substantial overlap between groups may occur, which reduces the discriminative capacity of purely geometric validation metrics. Therefore, the observed silhouette value should be interpreted as reflecting modest internal structure rather than strong cluster separation.

Repeated subsampling analysis showed moderate internal reproducibility of the selected five-cluster solution. Across 500 random subsamples containing 80% of the cohort, the mean ARI was 0.57 ± 0.14 and the median ARI was 0.54 (IQR: 0.46–0.63). The adjacent four-cluster solution showed comparable stability (mean ARI 0.57 ± 0.11; median 0.55, IQR: 0.50–0.63), whereas stability decreased for the six-cluster solution (mean ARI 0.46 ± 0.08; median 0.46, IQR: 0.40–0.51). Within the selected five-cluster solution, cluster-specific stability was heterogeneous, with mean Jaccard similarities of 0.66, 0.41, 0.51, 0.59, and 0.70 for Clusters S, E, O, F, and Y, respectively. These findings indicate moderate overall reproducibility, with greater stability for Clusters Y and S and lower stability for Cluster E.

**Figure 3:**
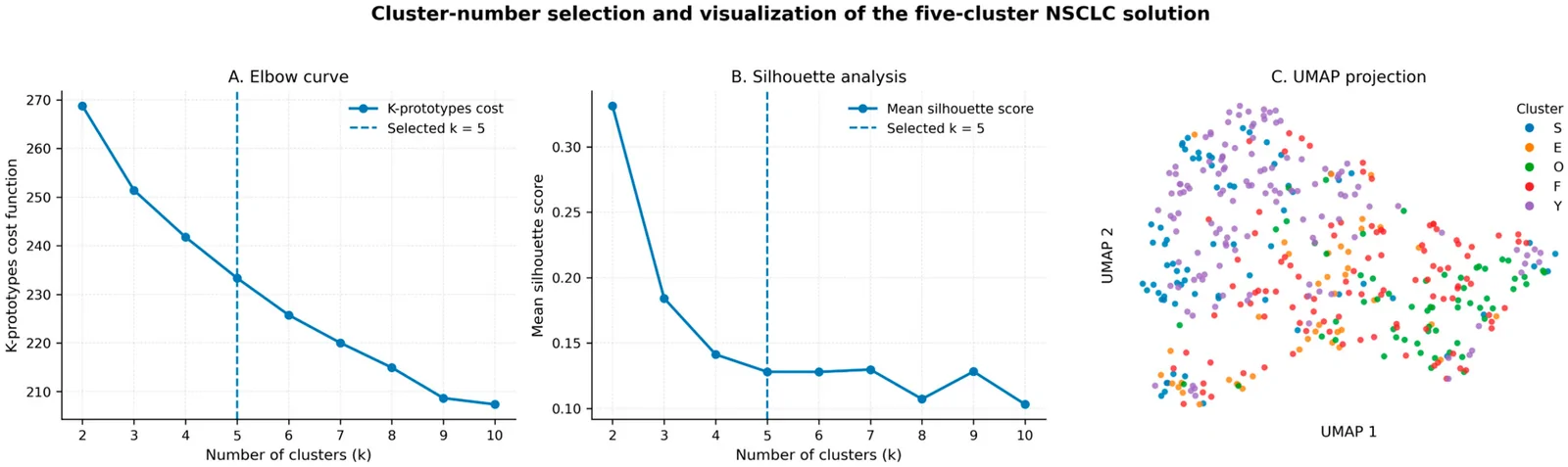
Cluster-number selection and visualization of the selected clustering solution. (**A**) K-prototypes cost function across candidate solutions from k = 2 to k = 10, used for elbow analysis. (**B**) Mean silhouette index across the same range of candidate clustering solutions. The dashed vertical line in panels A and B indicates the selected five-cluster solution. (**C**) Two-dimensional UMAP projection based on the Gower distance matrix for the selected five-cluster solution. Each point represents an individual patient and is coloured according to cluster assignment. The UMAP projection is shown for visualization purposes only.

To provide a more comprehensive visual assessment of the clustering solution, Figure 3 summarizes both the cluster-selection process and the final patient distribution. Panels A and B show the k-prototypes cost function and the mean silhouette index, respectively, across the candidate solutions from k = 2 to k = 10, with the selected five-cluster solution indicated by the dashed line. Panel C shows the two-dimensional UMAP projection based on the Gower distance matrix for the selected solution. The UMAP representation showed partial grouping according to cluster assignment, although with considerable overlap among clusters, consistent with the modest silhouette index. The UMAP projection was used exclusively for visualization and did not inform cluster selection.

**Figure 4:**
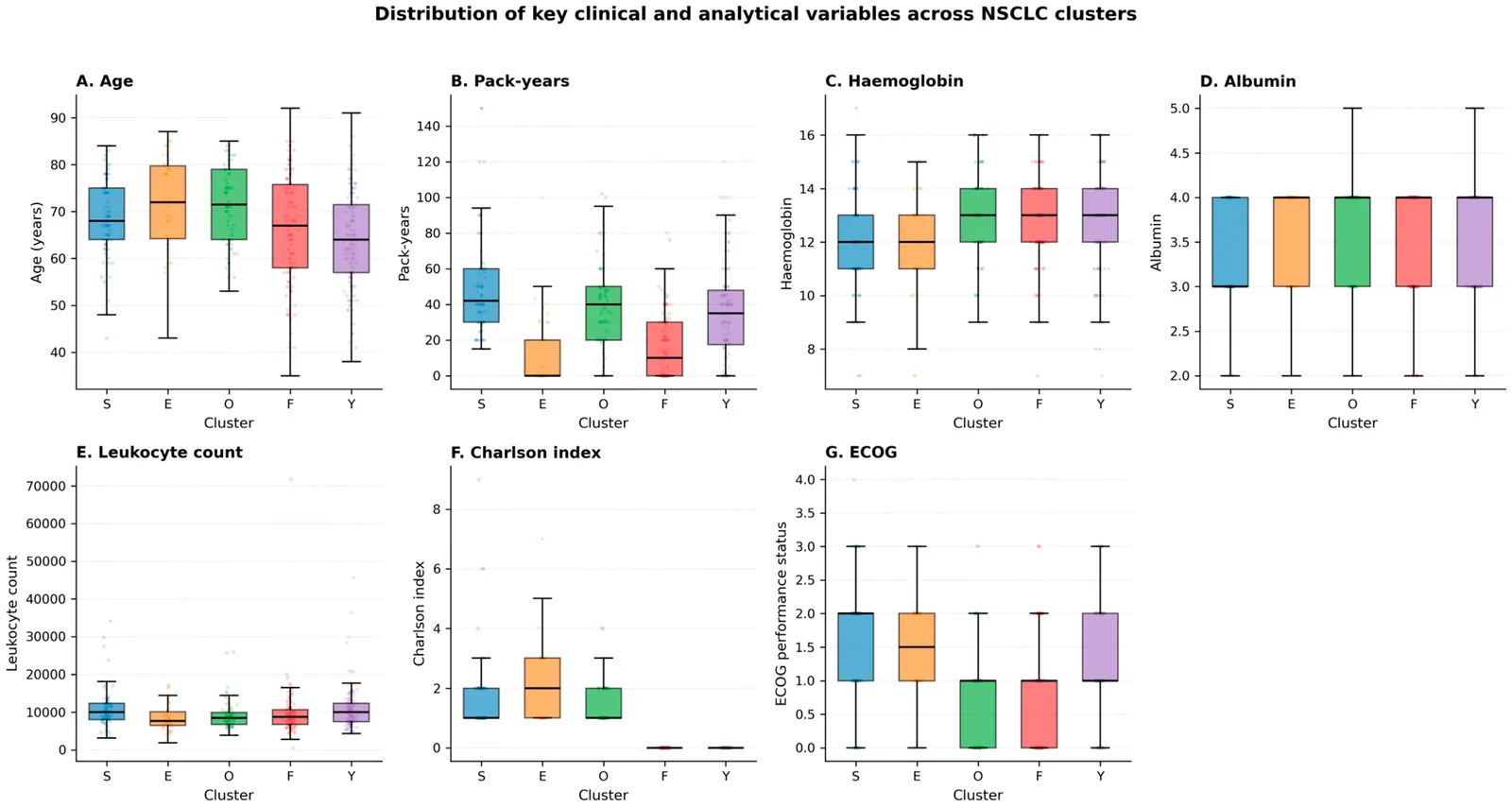
Distribution of key clinical and analytical variables across the five identified NSCLC clusters. Boxplots show the distributions of (**A**) age, (**B**) smoking exposure expressed as pack-years, (**C**) haemoglobin, (**D**) albumin, (**E**) leukocyte count, (**F**) Charlson Comorbidity Index, and (**G**) ECOG performance status. Boxes represent the interquartile range, central lines indicate the median, and individual observations are superimposed to illustrate within-cluster variability.

To further facilitate comparison of the clinical and analytical characteristics of the identified patient profiles, the distributions of selected continuous variables across clusters are shown in Figure 4. These graphical representations complement the descriptive and inferential results reported in Table 1 by illustrating both between-cluster differences and within-cluster variability.

### 3.2. Association of Clusters with NSCLC Biomarkers and Survival

Table 2 summarizes the association between cluster membership, molecular biomarker distribution, and survival outcomes. Molecular biomarkers were identified in 54% of the overall cohort. PD-L1 expression was the most common biomarker (34.75%), followed by EGFR mutation (14.5%). The overall mortality rate was 73.25%, and mean survival was 11.6 ± 17.3 months. Given the right-skewed distribution of survival times, median-based estimates are additionally reported in Table 3. The median observed survival time was 7.00 months (IQR: 2.00–16.00), while the Kaplan–Meier estimated median overall survival was 8.00 months (95% CI: 6.00–11.00).

Cluster F showed the highest prevalence of molecular biomarkers, mainly driven by EGFR mutations, and also exhibited the most favourable survival, approaching 17 months. In Cluster O, PD-L1 expression predominated, although mean survival remained below 12 months. Cluster E showed an intermediate biomarker prevalence (54.8%) without a clearly dominant molecular profile, and survival was also among the lowest, below one year. Cluster S showed a low prevalence of molecular alterations and one of the poorest survival outcomes. Cluster Y showed an intermediate prevalence of molecular biomarkers; PD-L1 expression was the most frequent biomarker, and the Kaplan–Meier median overall survival was 6 months. Kaplan–Meier median overall survival differed across clusters, ranging from 2.00 months (95% CI: 1.00–4.00) in Cluster S to 18.00 months (95% CI: 11.00–22.00) in Cluster F (Table 3).

**Figure 5:**
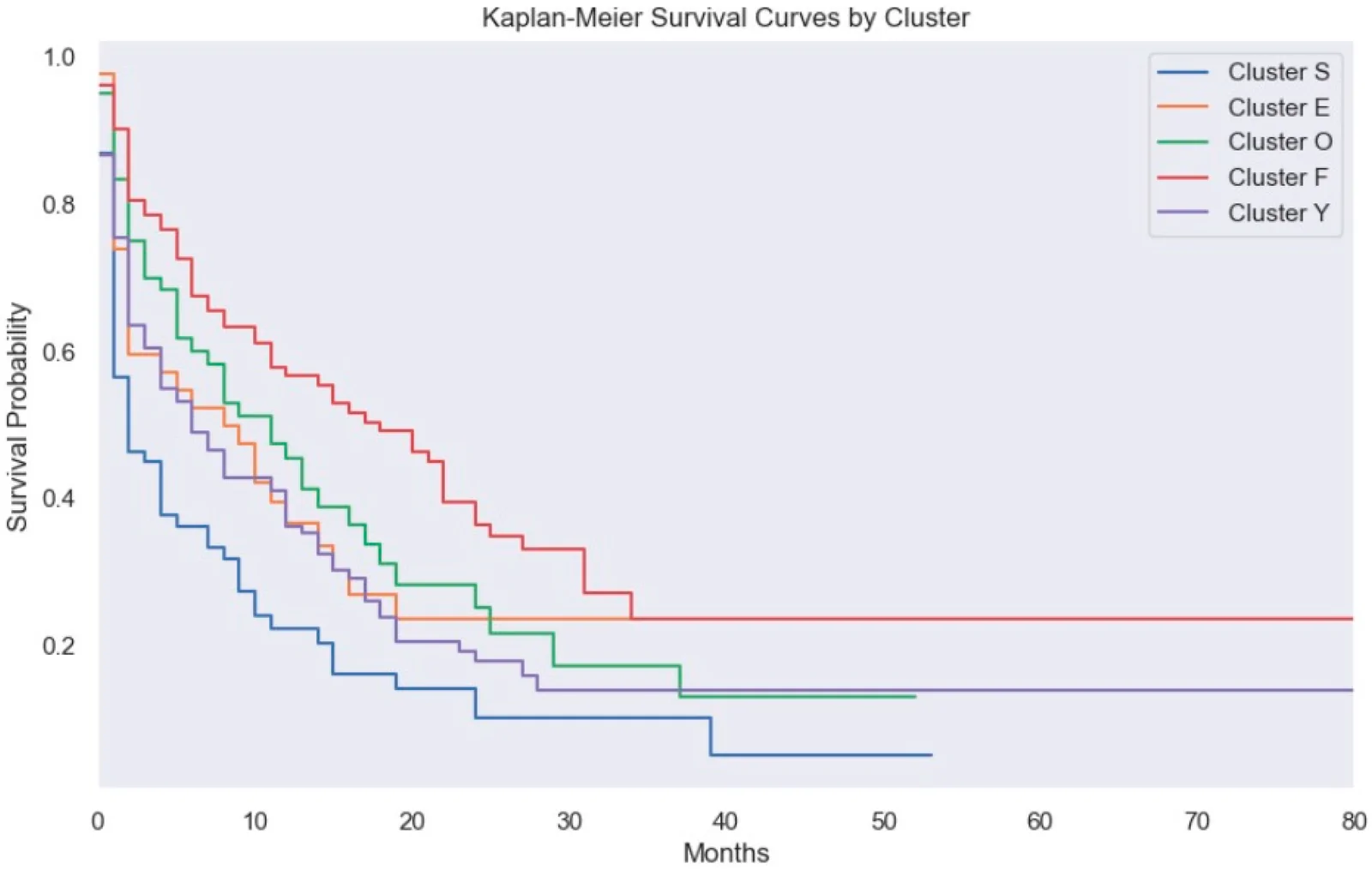
Kaplan–Meier overall survival curves stratified by cluster assignment. Survival distributions are shown for each cluster, with time expressed in months and overall survival as the outcome.

**Figure 6:**
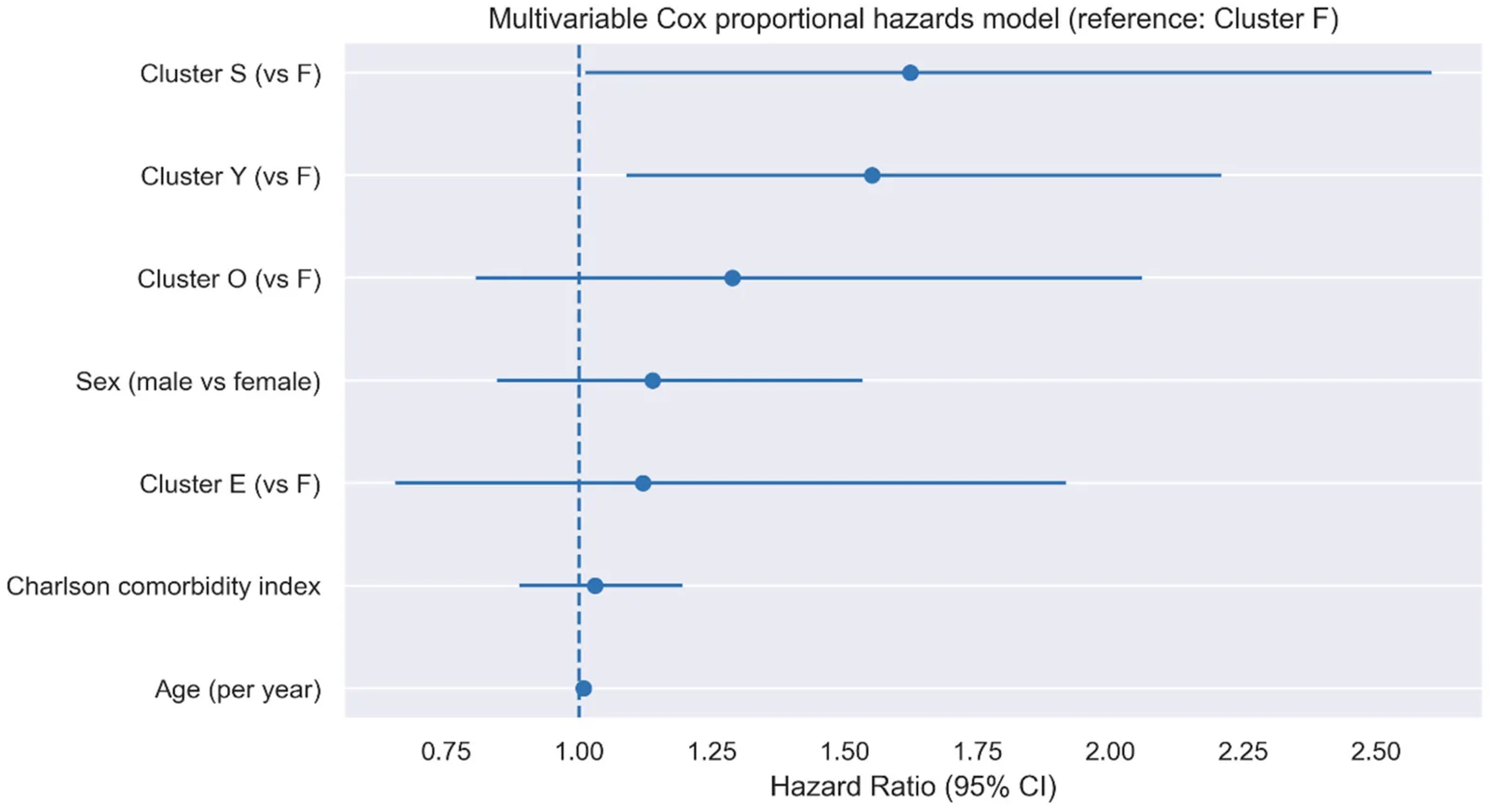
Results of the multivariable Cox proportional hazards model evaluating overall survival according to cluster assignment. Hazard ratios are shown for each cluster relative to the reference group (Cluster F), adjusted for age, sex, and Charlson comorbidity index, with ECOG included as a stratification variable.

Kaplan–Meier analysis showed visually distinct survival patterns across clusters (Figure 5). To further assess the association between cluster membership and overall survival, a multivariable Cox proportional hazards model was performed including cluster as a categorical variable and adjusting for age, sex, and Charlson comorbidity index. Schoenfeld residual-based diagnostics showed evidence of non-proportional hazards for ECOG performance status (test statistic = 9.22, *p* = 0.002), whereas no evidence of violation was observed for age, sex, Charlson comorbidity index, or any of the cluster contrasts (all *p* ≥ 0.319). ECOG was therefore incorporated as a stratification variable in the final Cox model. Reassessment of the proportional-hazards assumption in the final stratified model showed no evidence of violation for any of the remaining covariates (all *p* ≥ 0.435; Table A2). Taking Cluster F as the reference group, patients in Cluster S had a significantly higher risk of death (HR 1.62, 95% CI 1.01–2.60; *p* = 0.045), and patients in Cluster Y also had a significantly higher risk (HR 1.55, 95% CI 1.09–2.21; *p* = 0.015), whereas no significant differences were observed for Clusters E or O. These findings are visually summarized in Figure 6, while the complete numerical results of the multivariable Cox model, including the estimated effects of all covariates, are provided in Table A1.

## 4. Discussion

The clustering of patients with advanced-stage NSCLC using AI represents a relevant step toward more refined patient stratification. The application of unsupervised ML techniques enabled the identification of five patient profiles characterized by differences in clinical and analytical features. These clusters were consistent with patient profiles commonly observed in clinical practice and were associated with variations in molecular biomarker distribution and survival outcomes.

The contribution of this clustering approach should not be interpreted as the identification of novel individual prognostic factors or as a replacement for established clinical classifications. Rather, its value lies in integrating multiple demographic, clinical, analytical, comorbidity, and staging variables into multidimensional patient profiles that may complement conventional stratification. Although age and sex contributed to the differentiation of some clusters, the identified profiles were not defined by these variables alone. Whether cluster membership provides incremental prognostic information beyond established clinical and molecular factors requires dedicated comparative modelling and independent validation.

These findings should be interpreted in the context of the growing application of artificial intelligence in medicine. AI is increasingly being investigated across different pathologies [15,16,17,18,19,20,21,22,23,24], and recent reviews have highlighted its application in lung cancer, particularly in CT-based nodule detection and cytological-histological diagnosis [42,43]. Most applications, however, remain focused on imaging-based approaches and pulmonary nodule characterization [44], whereas comparatively few studies have explored patient-level stratification in advanced NSCLC using unsupervised learning approaches integrating clinical and analytical variables. In this context, the present study identified five patient profiles based on clinical, epidemiological, and analytical characteristics and examined their associations with molecular biomarkers and survival outcomes.

Cluster F was characterized by a predominance of women, relatively low smoking exposure, and a higher frequency of EGFR mutations. This finding is consistent with the increasing incidence of lung cancer in non-smoking women reported in recent decades [45]. In particular, studies in Asian populations have demonstrated a higher prevalence of EGFR mutations and other genetic factors that may contribute to this trend [46]. A similar pattern has been previously described in a Caucasian population by our group [4].

In contrast, Clusters S and O were characterized by a higher proportion of smokers. Continued smoking after NSCLC diagnosis has been associated with worse prognosis, increased adverse effects, reduced quality of life, and a higher risk of recurrence and second malignancies [47]. Identifying this patient profile may be useful for tailoring smoking cessation interventions, as quitting smoking after diagnosis has been shown to improve both survival and quality of life [5,47]. Both clusters also exhibited higher Charlson comorbidity indices, suggesting that smoking contributes to the burden of comorbid conditions such as COPD, which are themselves associated with poorer outcomes [48]. Additionally, smoking has been linked to differences in metastatic patterns [49], as reflected by the high prevalence of M1c disease in Cluster S. In Cluster O, the presence of additional exposure-related risk factors may further contribute to the observed prognosis [50].

Another distinguishing factor among clusters was leukocyte count. Both Clusters Y and S showed elevated values compared to the overall population. Previous studies have reported that tumor-related leukocytosis occurs in approximately 24% of patients with advanced NSCLC and is associated with poorer survival outcomes [51], which is consistent with our findings. In contrast, clusters with normal levels of proteins, albumin, and haemoglobin—such as Cluster F—demonstrated better prognosis [52]. Given that these parameters are routinely available in clinical practice, they may represent practical tools for patient stratification, as previously suggested [52].

The presence of pleural effusion was a defining characteristic of Cluster E. This feature is well known to be associated with worse prognosis in NSCLC [53]. Despite a relatively high rate of EGFR mutations, patients in this cluster showed shorter survival compared to Cluster F. Additionally, Cluster E had a higher mean age and a greater burden of comorbidities, similar to Cluster O, which may contribute to poorer outcomes by limiting treatment options and increasing the risk of complications [54].

Molecular biomarkers were deliberately excluded from cluster generation and were used instead for the subsequent characterization of the identified patient profiles. This allowed molecular differences to be assessed across clinically derived clusters without circularity. Consequently, the identified profiles primarily reflect clinical and phenotypic heterogeneity rather than an integrated clinical-molecular classification. Future studies incorporating molecular variables directly into the clustering process could provide complementary, biologically enriched patient profiles.

Regarding molecular characteristics, lower smoking exposure appeared to be associated with a higher frequency of driver mutations, as observed in Clusters E and F [55]. In contrast, Cluster Y was characterized by younger men with a higher prevalence of PD-L1 expression despite relatively poor survival. Previous studies have shown that PD-L1 expression does not consistently correlate with clinical variables or smoking status [47,56], although it may be associated with poorly differentiated tumors and more aggressive disease behavior [57].

Survival differences across clusters persisted after adjustment for age, sex, and Charlson Comorbidity Index, with ECOG incorporated as a stratification variable. However, because these variables also contributed to cluster derivation, the adjusted hazard ratios should not be interpreted as independent prognostic effects. The Cox model therefore provides an exploratory assessment of the association between the identified profiles and overall survival. Molecular and treatment-related variables were not included, while other cluster-defining prognostic characteristics, including tumor stage, metastatic features, and histology, were not additionally introduced as covariates to avoid further redundancy. Residual confounding therefore remains possible, and these findings should be considered exploratory and hypothesis-generating.

An additional limitation is the absence of treatment-related variables. Treatment selection and eligibility are major determinants of survival in advanced NSCLC, particularly for targeted therapies and immunotherapy, and may partly explain the observed differences across clusters. This is especially relevant for Cluster F, which showed a higher prevalence of EGFR mutations and the most favorable survival profile; the potential contribution of EGFR-targeted therapy cannot be assessed from the present data. Future studies incorporating detailed treatment information will be needed to determine whether these survival associations persist after accounting for therapeutic management.

One of the main limitations of this study is its single-centre and retrospective design. Although the cohort comprised 400 well-characterized patients with advanced-stage NSCLC, the identified profiles may reflect population- and setting-specific characteristics. The inclusion of patients for whom biomarker testing had been requested may also have introduced selection bias, potentially limiting the representativeness of the cohort. Accordingly, the findings should be considered exploratory rather than a clinically validated classification system. External validation in independent multicenter cohorts encompassing diverse populations and healthcare settings will be necessary to assess the reproducibility and generalizability of the identified profiles. Future studies should also explore their applicability in earlier disease stages.

Methodological limitations related to clustering should also be acknowledged. Internal validation of mixed-type clinical data remains challenging, and the relatively low Gower-based silhouette index indicates limited geometric separation between clusters, consistent with overlapping clinical phenotypes. As expected for an unsupervised approach, no predefined ground-truth labels were available against which the identified clusters could be benchmarked. Repeated subsampling showed that the five-cluster solution had stability comparable to the four-cluster solution, whereas partitioning into six clusters reduced reproducibility. Nevertheless, cluster-specific stability was heterogeneous, particularly for Cluster E, reinforcing the exploratory nature of these profiles.

In conclusion, this pilot study demonstrates the feasibility of unsupervised machine learning for identifying data-driven patient profiles in advanced-stage NSCLC. These profiles differed in clinical characteristics, molecular biomarkers, and overall survival, supporting the potential of this approach to characterize disease heterogeneity. Given the retrospective single-centre design and absence of external validation, however, the findings remain exploratory and require independent multicentre validation before their clinical utility can be established.

## Figures and Tables

**Table 1:** Cohort and cluster characteristics.

Variable	Population Summary	Cluster S (*n* = 69)	Cluster E (*n* = 42)	Cluster O (*n* = 60)	Cluster F (*n* = 102)	Cluster Y (*n* = 127)	Global Test	Global *p*-Value	Significant Pairwise Comparisons
Age	67.29 ± 10.67	68.72 ± 8.59	71.52 ± 10.35	71.30 ± 8.61	66.21 ± 11.89	64.09 ± 10.57	Kruskal–Wallis	≤0.001	S vs. Y: p_adj = 0.019; E vs. Y: p_adj = 0.001; O vs. Y: p_adj = ≤0.001
% Men	70.50% (65.85, 74.76)	88.41% (78.75, 94.01)	61.90% (46.81, 75.00)	85.00% (73.89, 91.90)	38.24% (29.39, 47.93)	82.68% (75.16, 88.27)	Chi-square	≤0.001	S vs. E: p_adj = 0.023; S vs. F: p_adj = ≤0.001; O vs. F: p_adj = ≤0.001; F vs. Y: p_adj = ≤0.001
Pack-years	31.26 ± 27.76	50.26 ± 28.88	11.83 ± 20.75	40.57 ± 24.96	16.14 ± 18.73	35.10 ± 26.70	Kruskal–Wallis	≤0.001	S vs. E: p_adj = ≤0.001; S vs. F: p_adj = ≤0.001; S vs. Y: p_adj = 0.004; E vs. O: p_adj = ≤0.001; E vs. Y: p_adj = ≤0.001; O vs. F: p_adj = ≤0.001; F vs. Y: p_adj = ≤0.001
% Risk factor: Yes	19.25% (15.69, 23.40)	23.19% (14.81, 34.40)	7.14% (2.46, 19.01)	26.67% (17.13, 39.01)	10.78% (6.13, 18.29)	24.41% (17.76, 32.56)	Chi-square	0.009	None significant after Bonferroni correction
% Symptoms: Yes	86.50% (82.80, 89.50)	82.61% (72.02, 89.76)	85.71% (72.16, 93.28)	76.67% (64.56, 85.56)	85.29% (77.15, 90.88)	94.49% (89.06, 97.30)	Chi-square	0.012	O vs. Y: p_adj = 0.008
Hemoglobin	12.62 ± 1.83	12.00 ± 1.96	12.02 ± 1.98	13.10 ± 1.71	12.73 ± 1.61	12.85 ± 1.82	Kruskal–Wallis	≤0.001	S vs. O: p_adj = 0.009; S vs. F: p_adj = 0.042; S vs. Y: p_adj = 0.008
Albumin	3.53 ± 0.60	3.36 ± 0.62	3.55 ± 0.55	3.67 ± 0.57	3.57 ± 0.61	3.51 ± 0.60	Kruskal–Wallis	0.042	S vs. O: p_adj = 0.040
Proteins	6.12 ± 0.75	6.16 ± 0.85	6.07 ± 0.84	6.17 ± 0.69	6.05 ± 0.67	6.14 ± 0.74	Kruskal–Wallis	0.706	-
Leukocytes	10,168.06 ± 5705.82	11,042.88 ± 5447.83	8680.86 ± 3689.68	9302.00 ± 4059.65	9772.06 ± 7107.70	10,911.81 ± 5677.80	Kruskal–Wallis	0.002	S vs. E: p_adj = 0.044; E vs. Y: p_adj = 0.047
Charlson	0.74 ± 1.14	1.74 ± 1.34	2.10 ± 1.27	1.48 ± 0.72	0.00 ± 0.00	0.00 ± 0.00	Kruskal–Wallis	≤0.001	S vs. F: p_adj = ≤0.001; S vs. Y: p_adj = ≤0.001; E vs. O: p_adj = 0.044; E vs. F: p_adj = ≤0.001; E vs. Y: p_adj = ≤0.001; O vs. F: p_adj = ≤0.001; O vs. Y: p_adj = ≤0.001
ECOG	1.19 ± 0.89	1.59 ± 0.91	1.48 ± 0.77	0.82 ± 0.81	0.90 ± 0.84	1.28 ± 0.84	Kruskal–Wallis	≤0.001	S vs. O: p_adj = ≤0.001; S vs. F: p_adj = ≤0.001; E vs. O: p_adj = ≤0.001; E vs. F: p_adj = 0.002; O vs. Y: p_adj = 0.004; F vs. Y: p_adj = 0.011
% Pleural effusion	29.00% (24.77, 33.63)	27.54% (18.39, 39.05)	64.29% (49.17, 77.01)	15.00% (8.10, 26.11)	29.41% (21.45, 38.87)	24.41% (17.76, 32.56)	Chi-square	≤0.001	S vs. E: p_adj = 0.003; E vs. O: p_adj = ≤0.001; E vs. F: p_adj = 0.002; E vs. Y: p_adj = ≤0.001
% T1A	1.50% (0.69, 3.23)	1.45% (0.26, 7.76)	2.38% (0.42, 12.32)	3.33% (0.92, 11.36)	0.98% (0.17, 5.35)	0.79% (0.14, 4.33)	Chi-square	0.697	-
% T1B	4.00% (2.48, 6.40)	5.80% (2.28, 13.98)	4.76% (1.32, 15.79)	5.00% (1.71, 13.70)	2.94% (1.01, 8.29)	3.15% (1.23, 7.82)	Chi-square	0.855	-
% T2A	12.00% (9.17, 15.55)	8.70% (4.05, 17.70)	11.90% (5.19, 25.00)	23.33% (14.44, 35.44)	15.69% (9.89, 23.97)	5.51% (2.70, 10.94)	Chi-square	0.006	O vs. Y: p_adj = 0.008
% T2B	8.75% (6.36, 11.93)	2.90% (0.80, 9.97)	11.90% (5.19, 25.00)	11.67% (5.77, 22.18)	11.76% (6.86, 19.45)	7.09% (3.77, 12.92)	Chi-square	0.221	-
% T3	19.25% (15.69, 23.40)	10.14% (5.00, 19.49)	21.43% (11.71, 35.94)	21.67% (13.12, 33.62)	31.37% (23.18, 40.91)	12.60% (7.91, 19.49)	Chi-square	0.002	S vs. F: p_adj = 0.022; F vs. Y: p_adj = 0.009
% T4	43.75% (38.97, 48.65)	60.87% (49.07, 71.52)	19.05% (9.98, 33.30)	28.33% (18.51, 40.77)	24.51% (17.19, 33.68)	65.35% (56.74, 73.07)	Chi-square	≤0.001	S vs. E: p_adj = ≤0.001; S vs. O: p_adj = 0.004; S vs. F: p_adj = ≤0.001; E vs. Y: p_adj = ≤0.001; O vs. Y: p_adj = ≤0.001; F vs. Y: p_adj = ≤0.001
% TX	10.75% (8.08, 14.17)	10.14% (5.00, 19.49)	28.57% (17.17, 43.57)	6.67% (2.62, 15.93)	12.75% (7.60, 20.59)	5.51% (2.70, 10.94)	Chi-square	≤0.001	E vs. Y: p_adj = 0.001
% N0	15.00% (11.83, 18.83)	10.14% (5.00, 19.49)	16.67% (8.32, 30.60)	21.67% (13.12, 33.62)	20.59% (13.88, 29.43)	9.45% (5.49, 15.79)	Chi-square	0.060	-
% N1	6.25% (4.27, 9.06)	1.45% (0.26, 7.76)	7.14% (2.46, 19.01)	13.33% (6.91, 24.17)	10.78% (6.13, 18.29)	1.57% (0.43, 5.56)	Chi-square	0.003	O vs. Y: p_adj = 0.028
% N2	25.25% (21.24, 29.73)	31.88% (22.09, 43.58)	26.19% (15.30, 41.07)	28.33% (18.51, 40.77)	30.39% (22.31, 39.90)	15.75% (10.43, 23.08)	Chi-square	0.051	-
% N3	40.00% (35.32, 44.87)	39.13% (28.48, 50.93)	21.43% (11.71, 35.94)	28.33% (18.51, 40.77)	24.51% (17.19, 33.68)	64.57% (55.93, 72.35)	Chi-square	≤0.001	S vs. Y: p_adj = 0.011; E vs. Y: p_adj = ≤0.001; O vs. Y: p_adj = ≤0.001; F vs. Y: p_adj = ≤0.001
% NX	13.50% (10.50, 17.20)	17.39% (10.24, 27.98)	28.57% (17.17, 43.57)	8.33% (3.61, 18.07)	13.73% (8.36, 21.73)	8.66% (4.91, 14.85)	Chi-square	0.011	E vs. Y: p_adj = 0.027
% M0	0.75% (0.26, 2.18)	1.45% (0.26, 7.76)	0.00% (0.00, 8.38)	0.00% (0.00, 6.02)	1.96% (0.54, 6.87)	0.00% (0.00, 2.94)	Chi-square	0.381	-
% M1a	20.50% (16.83, 24.73)	4.35% (1.49, 12.02)	59.52% (44.49, 72.96)	20.00% (11.83, 31.78)	29.41% (21.45, 38.87)	9.45% (5.49, 15.79)	Chi-square	≤0.001	S vs. E: p_adj = ≤0.001; S vs. F: p_adj = 0.001; E vs. O: p_adj = 0.001; E vs. F: p_adj = 0.014; E vs. Y: p_adj = ≤0.001; F vs. Y: p_adj = 0.002
% M1b	26.50% (22.41, 31.03)	8.70% (4.05, 17.70)	9.52% (3.77, 22.07)	70.00% (57.49, 80.10)	39.22% (30.30, 48.92)	11.02% (6.68, 17.65)	Chi-square	≤0.001	S vs. O: p_adj = ≤0.001; S vs. F: p_adj = ≤0.001; E vs. O: p_adj = ≤0.001; E vs. F: p_adj = 0.009; O vs. F: p_adj = 0.003; O vs. Y: p_adj = ≤0.001; F vs. Y: p_adj = ≤0.001
% M1c	52.25% (47.36, 57.10)	85.51% (75.34, 91.93)	30.95% (19.07, 46.03)	10.00% (4.66, 20.15)	29.41% (21.45, 38.87)	79.53% (71.69, 85.63)	Chi-square	≤0.001	S vs. E: p_adj = ≤0.001; S vs. O: p_adj = ≤0.001; S vs. F: p_adj = ≤0.001; E vs. Y: p_adj = ≤0.001; O vs. Y: p_adj = ≤0.001; F vs. Y: p_adj = ≤0.001
% Large cell carcinoma	1.00% (0.39, 2.54)	4.35% (1.49, 12.02)	2.38% (0.42, 12.32)	0.00% (0.00, 6.02)	0.00% (0.00, 3.63)	0.00% (0.00, 2.94)	Chi-square	0.021	None significant after Bonferroni correction
% Adenocarcinoma	87.50% (83.90, 90.39)	79.71% (68.78, 87.51)	92.86% (80.99, 97.54)	90.00% (79.85, 95.34)	92.16% (85.28, 95.97)	85.04% (77.81, 90.21)	Chi-square	0.092	-
% Adenosquamous carcinoma	1.50% (0.69, 3.23)	1.45% (0.26, 7.76)	0.00% (0.00, 8.38)	0.00% (0.00, 6.02)	2.94% (1.01, 8.29)	1.57% (0.43, 5.56)	Chi-square	0.559	-
% Squamous carcinoma	4.00% (2.48, 6.40)	4.35% (1.49, 12.02)	0.00% (0.00, 8.38)	5.00% (1.71, 13.70)	3.92% (1.54, 9.65)	4.72% (2.18, 9.92)	Chi-square	0.717	-
% Undifferentiated carcinoma	6.00% (4.06, 8.77)	10.14% (5.00, 19.49)	4.76% (1.32, 15.79)	5.00% (1.71, 13.70)	0.98% (0.17, 5.35)	8.66% (4.91, 14.85)	Chi-square	0.076	-

“Risk factor: Yes” indicates the presence of at least one recorded cancer-related risk factor or exposure other than smoking, which was considered separately. “Symptoms: Yes” indicates the presence of at least one clinical symptom at the time of diagnosis.

**Table 2:** Comparison of molecular biomarkers and survival rates across the five identified clusters.

Variable	Population Summary	Cluster S (*n* = 69)	Cluster E (*n* = 42)	Cluster O (*n* = 60)	Cluster F (*n* = 102)	Cluster Y (*n* = 127)	Global Test	Global *p*-Value	Significant Pairwise Comparisons
% Biomarkers presence	54.00% (49.10, 58.82)	44.93% (33.77, 56.62)	54.76% (39.95, 68.78)	51.67% (39.31, 63.82)	63.73% (54.05, 72.40)	51.97% (43.35, 60.47)	Chi-square	0.163	-
% ALK	2.75% (1.54, 4.86)	4.35% (1.49, 12.02)	4.76% (1.32, 15.79)	0.00% (0.00, 6.02)	1.96% (0.54, 6.87)	3.15% (1.23, 7.82)	Chi-square	0.508	-
% EGFR	14.50% (11.39, 18.29)	4.35% (1.49, 12.02)	23.81% (13.48, 38.53)	8.33% (3.61, 18.07)	32.35% (24.06, 41.93)	5.51% (2.70, 10.94)	Chi-square	≤0.001	S vs. F: p_adj = ≤0.001; E vs. Y: p_adj = 0.018; O vs. F: p_adj = 0.010; F vs. Y: p_adj = ≤0.001
% PD-L1	34.75% (30.25, 39.54)	36.23% (25.90, 48.02)	23.81% (13.48, 38.53)	41.67% (30.06, 54.27)	24.51% (17.19, 33.68)	42.52% (34.27, 51.21)	Chi-square	0.020	None significant after Bonferroni correction
% ROS1	1.25% (0.54, 2.89)	0.00% (0.00, 5.27)	2.38% (0.42, 12.32)	0.00% (0.00, 6.02)	2.94% (1.01, 8.29)	0.79% (0.14, 4.33)	Chi-square	0.325	-
% Death	73.25% (68.71, 77.35)	86.96% (77.03, 92.98)	71.43% (56.43, 82.83)	71.67% (59.23, 81.49)	61.76% (52.07, 70.61)	76.38% (68.28, 82.92)	Chi-square	0.006	S vs. F: p_adj = 0.006
Survival (months)	11.63 ± 17.31	6.99 ± 10.25	11.19 ± 15.06	11.72 ± 12.05	16.97 ± 26.07	9.97 ± 12.94	Kruskal–Wallis	≤0.001	S vs. O: p_adj = 0.007; S vs. F: p_adj = ≤0.001; F vs. Y: p_adj = 0.001

**Table 3:** Overall survival characteristics according to cluster assignment.

Group	N	Deaths, *n* (%)	Median Observed Survival, Months (IQR)	Kaplan–Meier Median Overall Survival, Months (95% CI)
Overall cohort	400	293 (73.2%)	7.00 (2.00–16.00)	8.00 (6.00–11.00)
Cluster S	69	60 (87.0%)	2.00 (1.00–9.00)	2.00 (1.00–4.00)
Cluster E	42	30 (71.4%)	7.00 (1.25–14.00)	8.00 (2.00–14.00)
Cluster O	60	43 (71.7%)	8.50 (2.75–15.25)	11.00 (5.00–16.00)
Cluster F	102	63 (61.8%)	11.00 (5.00–24.00)	18.00 (11.00–22.00)
Cluster Y	127	97 (76.4%)	6.00 (1.50–14.00)	6.00 (4.00–11.00)

IQR, interquartile range; CI, confidence interval. The median observed survival represents the descriptive median of the recorded survival times, whereas Kaplan–Meier median OS accounts for censored observations.

## Data Availability

The dataset is not publicly available as the research is ongoing. The data will be made available from the authors upon reasonable request.

## Appendix A

**Table A1:** Full multivariable Cox proportional hazards model for overall survival.

Variable	HR	95% CI	*p*-Value
Age, per year	1.01	1.00–1.02	0.127
Sex, male vs. female	1.14	0.85–1.53	0.393
Charlson Comorbidity Index, per point	1.03	0.89–1.20	0.698
Cluster S vs. F	1.62	1.01–2.60	0.045
Cluster E vs. F	1.12	0.65–1.92	0.680
Cluster O vs. F	1.29	0.81–2.06	0.290
Cluster Y vs. F	1.55	1.09–2.21	0.015

HR, hazard ratio; CI, confidence interval. Cluster F was used as the reference category. ECOG performance status was included as a stratification variable and therefore no regression coefficient, hazard ratio, or p-value was estimated for ECOG.

**Table A2:** Proportional-hazards diagnostics based on Schoenfeld residuals for the initial and final Cox models.

Variable	Initial Test Statistic	Initial *p*-Value	Final Test Statistic	Final *p*-Value
Age, per year	0.08	0.774	0.08	0.780
Sex, male vs. female	0.13	0.719	0.01	0.921
Charlson Comorbidity Index, per point	0.99	0.319	0.61	0.435
ECOG performance status	9.22	0.002	—	—
Cluster S vs. F	0.00	0.979	0.38	0.538
Cluster E vs. F	0.02	0.898	0.00	0.950
Cluster O vs. F	0.42	0.519	0.09	0.763
Cluster Y vs. F	0.17	0.680	0.02	0.880

A *p*-value < 0.05 was considered evidence against the proportional-hazards assumption. The initial model included ECOG performance status as a covariate. Because ECOG showed evidence of non-proportional hazards, it was used as a stratification variable in the final model and was therefore not retested as an individual covariate in that model. Cluster F was the reference category.
